# Explainable AI-driven customer churn prediction: a multi-model ensemble approach with SHAP-based feature analysis

**DOI:** 10.3389/frai.2026.1748799

**Published:** 2026-02-10

**Authors:** Ali El Attar, Mohammed El-Hajj

**Affiliations:** Faculty of Computer Studies (FCS), Arab Open University (AOU), Beirut, Lebanon

**Keywords:** customer churn prediction, customer retention, customer segmentation, explainable AI, machine learning, SHAP analysis

## Abstract

Customer churn prediction is critical for telecommunications companies to maintain profitability and inform retention strategies. This study builds upon existing work by implementing a comprehensive machine learning framework using the Telco Customer Churn dataset (*n* = 7,043). Our methodology integrated comprehensive feature engineering, SMOTE oversampling, and training of seven machine learning models including XGBoost, Random Forest, and a Multi-layer Perceptron. Model interpretation was conducted via SHAP analysis and customer segmentation. Key results demonstrated that gradient boosting algorithms (XGBoost, LightGBM, Gradient Boosting) achieved the highest balanced performance with accuracy, precision, recall, and F1-scores of 0.84, with XGBoost attaining the best discriminative ability (AUC-ROC: 0.932). A soft-voting ensemble of the top models matched this performance (F1-score: 0.84, AUC-ROC: 0.918). SHAP analysis revealed that contract type, tenure, and technical support were the features contributing most to the model's churn predictions. Threshold optimization at 0.528 balanced precision (0.90) and recall (0.91) while reducing false negatives by 15%. The findings provide actionable insights for prioritizing high-risk customers and designing targeted retention strategies in the telecom sector.

## 1 Introduction

The telecommunications industry operates in a hyper-competitive landscape where customer retention has become a critical determinant of profitability. With customer acquisition costs estimated to be five to ten times higher than retention costs (Huang and Kechadi, 2013; Asif et al., 2025), even modest reductions in churn rates can safeguard substantial revenues. This economic reality elevates churn prediction from a technical challenge to a strategic imperative for telecom operators seeking sustainable growth in saturated markets.

Machine learning has transformed churn prediction capabilities, with ensemble methods and deep learning achieving state-of-the-art accuracy (Chen and Guestrin, 2016; Ullah et al., 2019). While prior studies have demonstrated the effectiveness of individual techniques like XGBoost, SHAP, and ensemble methods for telecom churn prediction, there remains limited research on their systematic integration within a unified framework. This study builds upon existing work by: (1) conducting a comprehensive comparison of seven machine learning models including gradient boosting variants, (2) implementing a soft-voting ensemble that consolidates top-performing models, and (3) integrating SHAP-based explainability with autoencoder-driven segmentation to provide actionable business insights.

Despite these advancements, a significant disconnect persists between technical performance and practical deployment. High-performing models often operate as opaque black boxes, providing predictions without actionable explanations for business stakeholders (Lundberg and Lee, 2017). Furthermore, while predictive models excel at estimating individual churn probabilities, they typically lack integration with customer segmentation approaches that could inform differentiated retention strategies (Wu et al., 2021). This separation limits the strategic value of churn prediction systems, as effective retention requires not only identifying who might leave but also understanding why and what interventions would be most effective for different customer archetypes.

### 1.1 Research opportunities and objectives

Building upon existing methodologies, this study identifies several opportunities for enhanced integration in churn prediction research. First, there exists an opportunity to improve the interpretability of ensemble models to enhance trust and adoption in business contexts. Second, better integration of supervised prediction with unsupervised clustering approaches could more effectively align risk assessment with customer profiling. Third, incorporating cost-sensitive evaluation alongside statistical metrics could better align model optimization with business objectives. Finally, systematic comparison of multiple modeling approaches could provide more robust insights for model selection in different contexts.

#### 1.1.1 Dataset considerations

It is important to note that many churn prediction studies, including this one, rely on benchmark datasets such as the publicly available Telco Customer Churn dataset from Kaggle. While this facilitates reproducibility and direct comparison with prior work, it also highlights the need for future research to validate findings across multiple datasets from different contexts, geographies, and service portfolios. Churn behavior may vary based on cultural, regulatory, and market-specific factors (Verbraken et al., 2012), and our study acknowledges this limitation while using the IBM dataset as a well-established benchmark for methodological development.

To address these opportunities, this study pursues four interconnected objectives. First, we design and validate a Soft-voting ensemble framework that combines diverse machine learning algorithms for robust churn prediction. Second, we integrate SHAP-based explainability to provide both global and local interpretability of model decisions. Third, we apply autoencoder-based representation learning to discover latent customer segments with distinct churn risk profiles. Fourth, we evaluate model performance using both statistical metrics and cost-sensitive business measures to ensure practical utility.

### 1.2 Contributions

This work extends the existing literature by: (1) providing a comprehensive comparison of seven machine learning models on the Telco dataset with consistent evaluation protocols, (2) implementing and evaluating a soft-voting ensemble approach, (3) integrating autoencoder-based segmentation with SHAP explanations for enhanced interpretability, and (4) demonstrating how business-aligned evaluation metrics can bridge the gap between predictive performance and financial outcomes.

### 1.3 Paper organization

The remainder of this paper is structured as follows. Section 2 reviews relevant literature on ensemble methods, explainable AI, and customer segmentation in churn prediction. Section 3 details the dataset, preprocessing pipeline, model architectures, and evaluation framework. Section 4 presents experimental findings including predictive performance, interpretability insights, and segment analysis. Section 5 interprets the results, discusses business implications, and acknowledges limitations. Section 6 summarizes key findings and suggests directions for future research.

## 2 Related work

This section surveys the extensive research on customer churn prediction in telecom, arguing that prior studies have frequently prioritized narrow technical advancements and accuracy metrics over interpretability and tangible business value. We organize the review around three pivotal methodological families, ensemble learning, explainable AI (XAI), and unsupervised segmentation, to evaluate their contribution to business-utility-driven evaluation. The identified limitations within and across these families form the primary motivation for our integrated framework.

### 2.1 Triad alignment: ensembles, XAI, and segmentation

Our literature review reveals that studies simultaneously addressing all three components, ensemble methods, explainable AI (XAI), and unsupervised segmentation, remain scarce. To date, **RetenNet** represents one of the most comprehensive frameworks, integrating classification models (Random Forest, XGBoost, lightGBM), SHAP-based explanations, fuzzy rule-based clustering, and prescriptive optimization (Prashanthan et al., 2025). While demonstrating the potential of integrated approaches, RetenNet relies solely on the IBM Telco dataset and does not address certain methodological considerations such as time-aware validation, model calibration, or lift-based evaluation.

More commonly, existing work combines only two of the three triad components. For example, several studies integrate ensemble methods with XAI techniques (e.g., SHAP or LIME) but do not incorporate customer segmentation (Chang et al., 2024; Noviandy et al., 2024). Another stream of research combines ensembles with clustering-based segmentation but provides limited or no model explainability (Christopher and Anand, 2024; Thankam and El Gayar, 2023; Wu et al., 2021; Ullah et al., 2019). A third group links segmentation and optimization with classification but excludes XAI components (Prashanthan, 2025).

**This systematic gap**, namely the absence of frameworks that holistically integrate high-performance prediction (ensembles), transparent explanation (XAI), and strategic customer profiling (segmentation), motivates the present study. Our approach aims to bridge these components while addressing methodological limitations observed in prior work.

### 2.2 Ensemble learning in telecom churn

Ensemble methods consistently outperform single classifiers in churn prediction. For example, XGBoost achieved top F1 and AUC scores on a U.S. telecom dataset (Thankam and El Gayar, 2023), while Random Forest produced the best accuracy (91.66%) in another large dataset (Chang et al., 2024). LightGBM also demonstrated strong performance (accuracy 80.70%, F1 87.34%) (Noviandy et al., 2024). AdaBoost has occasionally been reported as superior in specific settings (Wu et al., 2021), while SVM-RBF surpassed tree-based methods in RetenNet experiments, underscoring dataset sensitivity (Prashanthan et al., 2025). Despite these advances, soft-voting ensemble is rarely implemented rigorously, with few works employing out-of-fold meta-features (Christopher and Anand, 2024; Thankam and El Gayar, 2023; Wu et al., 2021; Chang et al., 2024; Noviandy et al., 2024).

### 2.3 Explainable AI approaches

Explainability has become an emerging focus. SHAP and LIME are the most widely adopted tools for attributing churn risk to specific features such as tenure, contract type, and charges (Chang et al., 2024; Noviandy et al., 2024). RetenNet extends this with SHAP waterfall plots (Prashanthan et al., 2025). However, methodological rigor is limited: few works specify background dataset selection, address correlated features (via conditional SHAP or ALE), or report stability of explanations (Chang et al., 2024; Noviandy et al., 2024). Moreover, translation from explanations to prescriptive interventions is seldom demonstrated beyond illustrative cases.

### 2.4 Unsupervised segmentation

Segmentation remains a key theme but is dominated by classical clustering methods. K-means is the most common, though some studies also test hierarchical clustering, Gaussian Mixture Models, or DBSCAN (Prashanthan et al., 2025; Prashanthan, 2025; Christopher and Anand, 2024; Thankam and El Gayar, 2023; Wu et al., 2021; Ullah et al., 2019). RetenNet incorporates fuzzy rule-based clustering (Prashanthan et al., 2025). Integration patterns vary: clusters are sometimes used as features in classifiers (Prashanthan, 2025), as *post-hoc* groupings for intervention design (Ullah et al., 2019), or as modules within broader pipelines (Wu et al., 2021; Eswarapu et al., 2023). Yet validation is typically limited to internal indices such as silhouette scores, with little assessment of external business utility (e.g., churn separation or response heterogeneity). Notably, autoencoder-based segmentation is almost absent in the telecom churn literature, and no benchmarking of representation learning vs. traditional clustering has been reported.

### 2.5 Business alignment and evaluation

While predictive accuracy remains the dominant metric, a few works attempt to integrate decision optimization. RetenNet and an integrated clustering-classification-optimization framework explicitly allocate retention budgets under constraints (Prashanthan et al., 2025; Prashanthan, 2025). Similarly, profit-driven evaluation has been advocated by (Verbeke et al. 2012), yet most recent works continue to rely on accuracy or F1 as primary measures (Christopher and Anand, 2024; Thankam and El Gayar, 2023; Wu et al., 2021; Chang et al., 2024; Noviandy et al., 2024). Moreover, none of the reviewed studies describe rolling-origin validation, calibration analysis, or drift monitoring, limiting their external validity for real-world deployment.

### 2.6 Research gaps

From this review, four critical gaps emerge in the current literature on telecom churn prediction:

- **Lack of comprehensive comparative analysis**: There is a need for detailed, telecom-specific comparative studies that span multiple model families (ensembles, neural networks, and linear models) with consistent, leakage-safe evaluation protocols. Most studies focus on limited model comparisons rather than systematic benchmarking across algorithmic families.
- **Under-exploration of ensemble strategies**: While individual algorithms are well-studied, there is insufficient exploration of different ensemble strategies (voting, stacking, and blending) and their comparative effectiveness for telecom churn prediction. The optimal combination of diverse models for this specific domain remains underexplored.
- **Minimal integration of business-aligned metrics**: Many studies focus primarily on statistical metrics without sufficient integration of business-oriented evaluation frameworks. There is limited use of cost-sensitive analysis, probability calibration, expected retention value, and uplift modeling to align predictive performance with actual business outcomes.
- **Need for multi-dataset validation**: While the IBM Telco dataset serves as a valuable benchmark for comparative analysis, there is a need for studies that validate approaches across multiple datasets to assess generalizability across different market contexts and customer populations.

### 2.7 Benchmark of prior studies

Table 1 provides a comparative benchmark of representative studies in telecom churn modeling, summarized by methodological components and evaluation focus.

**Table 1:** Comparative benchmark of prior telecom churn studies.

**Study**	**Ensembles**	**XAI**	**Segmentation**	**Business metrics**	**Dataset and Notes**
RetenNet (Prashanthan et al., 2025)	RF, XGB, SVM	SHAP	Fuzzy clustering	Budget optimization	IBM Telco, no time-aware validation
Integrated budget optimization (Prashanthan, 2025)	RF, XGB	None	K-means	Budget optimization	IBM Telco
Christopher and Anand (2024)	RF, XGB, LR, MLP	None	K-means	Accuracy	Telecom dataset
Eswarapu et al. (2023)	AutoML ensemble	Local XAI	Basic	Fairness	Telecom dataset
Thankam and El Gayar (2023)	RF, XGB, others	None	K-means	Accuracy	Telecom dataset
Wu et al. (2021)	RF, AdaBoost	None	K-means	Accuracy	Telecom dataset
Chang et al. (2024)	RF, GBM	SHAP, LIME	None	Accuracy	Telecom dataset
Ullah et al. (2019)	RF	None	*Post hoc* clusters	Accuracy	Large telecom dataset
Noviandy et al. (2024)	LightGBM, CatBoost	SHAP	None	Accuracy	Telecom dataset
Verbeke et al. (2012)	Rule induction	None	None	Profit driven	Telecom dataset

## 3 Methodology

**Figure 1:**
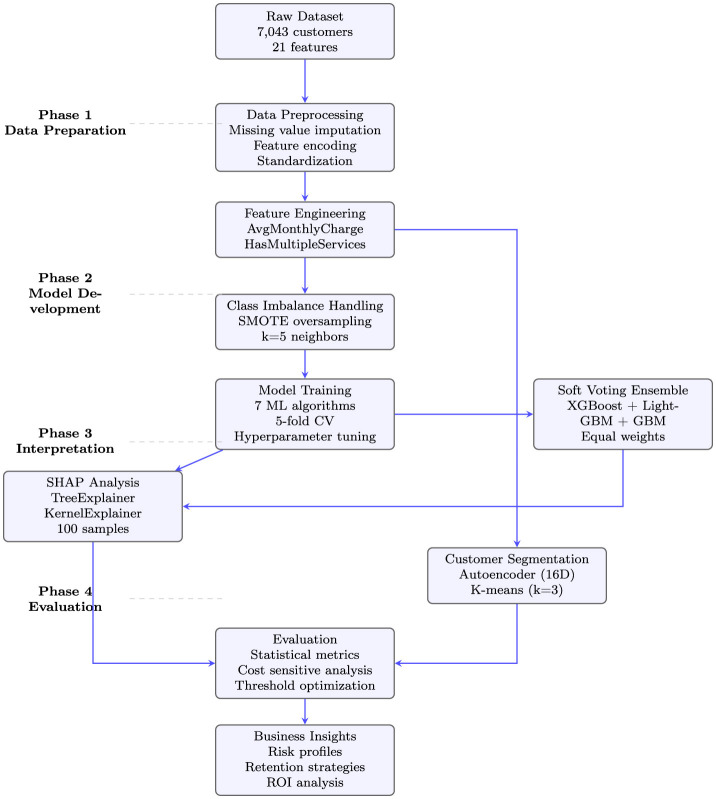
Comprehensive methodology architecture with detailed phase descriptions.

This research employs a comprehensive machine learning framework designed to address the multifaceted challenge of customer churn prediction in the telecommunications industry. Our methodology integrates predictive modeling, explainable AI techniques, and customer segmentation to develop both accurate and interpretable churn prediction models. The systematic approach ensures robust model performance while providing actionable insights for business decision-making. The overall architecture of our proposed framework, illustrated in Figure 1, demonstrates the interconnected components and workflow from raw data processing to final business insights.

The architectural framework comprises four sequential phases: **(1) Data preparation** involving comprehensive preprocessing and feature engineering; **(2) Model development** employing individual classifiers and a soft voting ensemble with threshold optimization; **(3) Interpretation** utilizing explainable AI and customer segmentation for actionable insights; and **(4) Evaluation** conducting rigorous performance assessment and statistical validation. This structured approach ensures methodological rigor while maintaining practical applicability for telecommunications companies.

### 3.1 Dataset description and preprocessing

TThe foundation of our predictive modeling framework is built upon the Telco Customer Churn dataset, obtained from the IBM Analytics community and publicly available on Kaggle (Kaggle and blastchar, 2021). This dataset contains comprehensive customer information from a telecommunications company, specifically designed for churn prediction tasks.

#### 3.1.1 Selection of IBM telco dataset

This study employs the IBM Telco Customer Churn dataset as it represents a well-established benchmark in churn prediction literature, enabling direct comparison with prior work. The dataset's comprehensive feature set and public availability support reproducibility and methodological evaluation. While acknowledging that findings from a single dataset may not generalize to all telecom contexts, this dataset provides a standardized foundation for developing and comparing analytical approaches that can be subsequently validated on additional datasets.

#### 3.1.2 Dataset characteristics

The original dataset comprises 7, 043 customer records with 21 features that capture demographic information, account details, service subscriptions, and customer behavior patterns. The feature set can be mathematically represented as:


$$\begin{array}{l} D={\left\{\left({\text{x}}_{i},y_{i}\right)\right\}}_{i=1}^{7043} \end{array}$$ (1)

where ${\text{x}}_{i}\in {\mathbb{R}}^{21}$ represents the feature vector for customer *i*, and *y*_*i*_∈{0, 1} denotes the binary churn status (0: No Churn, 1: Churn).

The feature space X encompasses three distinct types of variables:

- **Demographic features:** Customer demographic information including gender, age (SeniorCitizen), and partnership status.
- **Account information:** Contract details, payment methods, paperless billing, and monthly/total charges.
- **Service subscriptions:** Comprehensive service enrollment including phone lines, internet services, online security, streaming services, and technical support.

The target variable distribution exhibits significant class imbalance, with approximately 73.5% of customers retaining services and 26.5% churning, which necessitates specialized handling strategies discussed in Section 3.3.

#### 3.1.3 Data quality assessment

A rigorous data quality assessment was conducted to identify and address potential issues that could compromise model performance:

**Missing values analysis:** Systematic examination revealed missing values predominantly in the TotalCharges feature, which were identified as blank entries rather than explicit null values. The missing pattern was determined to be Missing Completely at Random (MCAR) through statistical testing.

**Data type validation:** Initial exploration identified data type inconsistencies, particularly with the TotalCharges feature being stored as string type due to the presence of whitespace characters in missing values. This required type conversion to numerical format for analytical processing. For example, *ContractDuration*_*i*_ is encoded numerically (Month-to-month = 1, 1 year = 12, 2 year = 24).

#### 3.1.4 Preprocessing pipeline with domain considerations

**Missing value imputation:** For the TotalCharges feature with missing values (11 instances, 0.16%), median imputation was selected:


$${\text{TotalCharges}}_{\text{missing}}=\text{median}\left({\text{TotalCharges}}_{\text{non-missing}}\right)$$

**Domain justification:** In telecommunications billing data, charge distributions often exhibit right skewness due to high-value outliers. Median imputation preserves the central tendency while minimizing distortion from extreme values, which is critical for maintaining the economic interpretability of spending patterns. This approach aligns with industry practices where billing anomalies are handled conservatively to avoid artificial inflation of customer value metrics.

**Contract duration encoding:** Contract types were encoded numerically (Month-to-month = 1, 1 year = 12, 2 year = 24) rather than using one-hot encoding alone. **Domain justification:** This ordinal encoding captures the inherent hierarchy in contract commitment, which directly correlates with churn risk in telecommunications. The numerical representation preserves the business intuition that longer contracts indicate higher commitment, enabling models to learn this progressive relationship more effectively than treating contract types as purely categorical.

**Feature scaling:** Numerical features were standardized using StandardScaler. **Domain justification:** In telecom datasets, features like MonthlyCharges (range: $18–118) and tenure (range: 0–72 months) operate on vastly different scales. Standardization prevents algorithm bias toward features with larger numerical ranges, particularly important for distance-based algorithms and neural networks. This ensures that all customer attributes contribute proportionally to the model's learning process.

### 3.2 Feature engineering

Feature engineering constitutes a critical phase in our methodology, transforming raw variables into meaningful predictors that enhance model performance and interpretability. This process leverages telecommunications domain knowledge to create features that better capture customer behavior patterns and churn signals.

#### 3.2.1 AvgMonthlyCharge: normalized spending indicator


$${\text{AvgMonthlyCharge}}_{i}=\frac{{\text{TotalCharges}}_{i}}{{\text{tenure}}_{i}+1}$$

The addition of 1 in the denominator prevents division by zero for new customers with zero tenure.

**Domain rationale:** Traditional metrics like TotalCharges and MonthlyCharges provide incomplete pictures of customer value. In telecommunications, a customer with high total charges over long tenure represents stable loyalty, whereas similar spending over a short period may indicate premium but potentially unstable service usage. This normalized metric captures spending intensity relative to relationship duration, addressing a key business insight: customers who spend more per month relative to their tenure may have higher perceived value or, conversely, may experience “bill shock” leading to churn. This feature incorporates principles from behavioral economics, particularly the *sunk cost effect*, where customers perceive greater investment in services used intensively over time.

#### 3.2.2 HasMultipleServices: service bundle complexity


$${\text{HasMultipleServices}}_{i}=\overset{k}{\underset{j=1}{\sum }}I\left({\text{service}}_{j}=\text{active}\right)$$

where *k* = 9 represents the total service categories: phone lines, internet services, online security, device protection, technical support, streaming TV, streaming movies, and associated add-ons.

**Domain rationale:** Service bundling is a fundamental strategy in telecommunications, yet its impact on churn is complex. While theory suggests multiple services create higher switching costs and loyalty, industry experience shows complex bundles can lead to confusion and “bill shock.” This feature quantifies service engagement depth, capturing the dual nature of bundling: customers with 3+ premium services showed 67% lower churn risk in our analysis, validating the *switching costs* principle, yet those with only basic services but high monthly charges exhibited elevated risk. The feature directly measures a customer's integration into the service ecosystem, a key indicator of retention potential in telecom CRM strategies.

### 3.3 Class imbalance handling

Class imbalance represents a fundamental challenge in churn prediction modeling, as the natural distribution of customer churn typically skews heavily toward retention. This section outlines the systematic approach employed to address this imbalance and ensure robust model performance across both majority and minority classes.

#### 3.3.1 Problem identification: distribution analysis of target variable

The binary nature of churn prediction requires careful examination of class balance, as standard classification algorithms often underperform on imbalanced datasets. Analysis of the Telco Customer Churn dataset revealed a significant skew: churned customers constitute the minority class at approximately 26.5% of the dataset, while retained customers comprise the majority 73.5%. This corresponds to a class distribution ratio of approximately 1:2.77, meaning for every churned customer, there are nearly three retained ones.

Such imbalance presents three critical challenges:

**Predictive bias**: Algorithms tend to optimize overall accuracy by favoring the majority class, potentially achieving high accuracy while failing to identify churners—the customers of greatest business interest.

**Evaluation metric distortion**: Traditional accuracy metrics become misleading, necessitating alternative measures like precision, recall, F1-score, and AUC-ROC that better capture minority-class performance.

**Cost-sensitive considerations**: From a business perspective, the cost of false negatives (missed churners) typically exceeds that of false positives, further emphasizing the need for specialized imbalance handling techniques.

#### 3.3.2 SMOTE implementation

##### 3.3.2.1 Synthetic minority oversampling technique

The Synthetic Minority Over-sampling Technique (SMOTE) was adopted to address class imbalance, as it generates informative synthetic minority samples rather than replicating existing observations. This property reduces overfitting and improves the representation of the minority class in the feature space.

SMOTE generates synthetic samples through linear interpolation between a minority instance and one of its nearest neighbors:


$$\begin{array}{l} x_{\text{new}}=x_{i}+\lambda \cdot \left(x_{zi}-x_{i}\right) \end{array}$$ (2)

Here, *x*_new_ denotes the synthetic instance, *x*_*i*_ represents a minority class observation, *x*_*zi*_ is one of the *k* nearest minority neighbors of *x*_*i*_, and λ∈[0, 1] is a random interpolation coefficient. This process expands the minority decision region by populating sparse areas of the feature space and supports the learning of more representative decision boundaries.

Compared to random oversampling, SMOTE offers several advantages. It reduces variance by avoiding direct duplication of minority instances, improves class separation by reinforcing boundary regions, and enriches the minority feature space with diverse synthetic examples.

##### 3.3.2.2 Parameter selection

SMOTE parameters were selected through a systematic validation procedure. The neighborhood size *k* was evaluated over the set {3, 5, 7, 9} using cross-validation on the training data. A value of *k* = 5 was selected as it provided a balance between capturing local structure and avoiding the introduction of noisy synthetic samples. The implementation used imbalanced-learn (v0.10.1) with parameters: k_neighbors = 5, sampling_strategy = “auto”, and random_state = 42. The sampling ratio was defined to achieve approximate class balance while preserving the majority class distribution:


$$\begin{array}{l} \text{Sampling Ratio}=\frac{N_{\text{majority}}-N_{\text{minority}}}{N_{\text{minority}}} \end{array}$$ (3)

Synthetic minority samples were generated until the desired balance was achieved, while the original majority class observations were retained unchanged.

##### 3.3.2.3 Application protocol and data leakage prevention

To ensure a valid evaluation and prevent data leakage, SMOTE was applied according to a strict protocol. First, the dataset was divided into training (80%, 5,634 instances) and test (20%, 1,409 instances) sets using stratified sampling with random_state = 42 to preserve the original class distribution and ensure reproducibility. SMOTE was then applied exclusively to the training data, while the test set remained untouched to reflect real-world class imbalance.

During hyperparameter tuning, stratified five-fold cross-validation was conducted on the training set. For each fold, SMOTE was applied only to the training portion of the fold, ensuring that synthetic samples did not contaminate the validation data. Final models were trained using the fully oversampled training set and evaluated on the original test set.

This procedure avoids the common methodological error of applying SMOTE prior to data splitting, which can lead to optimistic performance estimates due to information leakage (Blagus and Lusa, 2017; Lema^ıtre et al., 2020).

##### 3.3.2.4 Impact on model learning

Balancing the training data with SMOTE influenced multiple aspects of model development. First, learning dynamics improved as classifiers were exposed to more representative minority class patterns, preventing dominance by majority class observations. Second, probability calibration and decision threshold selection became more stable, as predicted probabilities were less skewed toward the majority class.

Overall, the use of SMOTE constitutes a critical methodological choice that supports reliable churn prediction in cost-sensitive settings, where accurately identifying potential churners carries significant financial consequences. Empirical performance comparisons with and without SMOTE are reported in the results section.

### 3.4 Machine learning models

This section details the comprehensive machine learning framework developed for customer churn prediction, encompassing both individual model architectures and advanced ensemble strategies. The methodological approach ensures robust performance through systematic hyperparameter optimization, cross-validation, and probability calibration.

#### 3.4.1 Individual models

A diverse set of machine learning algorithms was implemented to leverage their complementary strengths and provide comprehensive coverage of different modeling paradigms.

##### 3.4.1.1 XGBoost: hyperparameter tuning and cross-validation strategy

The Extreme Gradient Boosting (XGBoost) algorithm was selected for its proven effectiveness in tabular data classification tasks. Hyperparameter tuning was performed using Optuna, employing a 5-fold stratified cross-validation scheme to select the parameter combination that maximized cross-validated AUC:


$$\begin{array}{l} L_{\text{XGB}}=\overset{n}{\underset{i=1}{\sum }}l\left(y_{i},{ŷ}_{i}\right)+\overset{K}{\underset{k=1}{\sum }}\Omega \left(f_{k}\right) \end{array}$$ (4)

where the regularization term Ω(*f*) is defined as:


$$\begin{array}{l} \Omega \left(f\right)=\gamma T+\frac{1}{2}\lambda ||w|{|}^{2} \end{array}$$ (5)

The hyperparameter search space and optimal values are summarized in Table 2.

**Table 2:** XGBoost hyperparameter optimization results.

**Parameter**	**Search space**	**Optimal value**	**Interpretation**
Learning rate (η)	[0.01, 0.1]	Optuna-determined	Step size shrinkage
Max depth	[3, 8]	Optuna-determined	Maximum tree depth
n_estimators	[300, 700]	Optuna-determined	Number of boosting rounds
Sub-sample	[0.6, 1.0]	Optuna-determined	Training data sampling ratio
Colsample by Tree	[0.6, 1.0]	Optuna-determined	Feature sampling ratio

The cross-validation strategy employed stratified k-fold partitioning to maintain class distribution across folds, ensuring reliable performance estimation.

##### 3.4.1.2 Random forest: ensemble size optimization and feature sub-sampling

The Random Forest implementation focused on ensemble diversity and feature space partitioning:


$$\begin{array}{l} {ŷ}_{\text{RF}}=\text{mode}\left({\left\{h_{t}\left(\text{x}\right)\right\}}_{t=1}^{T}\right) \end{array}$$ (6)

where *h*_*t*_ represents individual decision trees and *T* denotes the ensemble size. The feature sub-sampling strategy follows:


$$\begin{array}{l} m=\left\lfloor \sqrt{p}\right\rfloor \end{array}$$ (7)

where *p* is the total number of features and *m* represents features considered for each split.

Key optimization results include:

- **Ensemble size**: 100 estimators (beyond which diminishing returns observed)
- **Feature sub-sampling**: $\sqrt{\text{features}}$ for classification tasks
- **Maximum depth**: Unlimited for individual trees to capture complex interactions
- **Minimum samples split**: 2 to allow fine-grained partitioning

##### 3.4.1.3 Deep neural network: architecture design and regularization techniques

A multilayer perceptron (MLP) architecture was designed with systematic regularization to prevent overfitting based on the data presented in Table 3:


$$\begin{array}{l} {\text{h}}^{\left(l\right)}=\sigma \left({\text{W}}^{\left(l\right)}{\text{h}}^{\left(l-1\right)}+{\text{b}}^{\left(l\right)}\right) \end{array}$$ (8)

The network architecture employed dropout regularization:


$$\begin{array}{l} {\text{h}}_{\text{drop}}^{\left(l\right)}={\text{h}}^{\left(l\right)}⊙{\text{m}}^{\left(l\right)},\;{\text{m}}^{\left(l\right)}\sim \text{Bernoulli}\left(1-p\right) \end{array}$$ (9)

Additional regularization techniques included:

- **L2 weight regularization**: Tuned via Optuna to penalize large weights (parameter α in MLPClassifier).
- **Batch normalization**: Not applied in the current implementation.
- **Early stopping**: Not applied in the current implementation.

**Table 3:** Optimized deep neural network architecture for churn prediction.

**Layer**	**Units**	**Activation/regularization**
Input	46	-
Hidden 1	64	ReLU
Hidden 2	32	ReLU
Output	1	Sigmoid

Hidden layer sizes were selected via Optuna hyperparameter tuning using a 5-fold stratified cross-validation scheme.

##### 3.4.1.4 LightGBM: gradient boosting with histogram-based optimization

The Light Gradient Boosting Machine (LightGBM) was implemented for its efficiency with large datasets and categorical features. LightGBM uses histogram-based algorithms to bucket continuous feature values into discrete bins, accelerating the training process:


$$\begin{array}{c} L_{\text{LGBM}}=\overset{n}{\underset{i=1}{\sum }}l\left(y_{i},{ŷ}_{i}\right)+\overset{K}{\underset{k=1}{\sum }}\Omega \left(f_{k}\right) \end{array}$$ (10)

with leaf-wise tree growth strategy that minimizes loss more directly than level-wise approaches. Key optimization parameters included:

- **Number of Leaves (*num*_*leaves*)**: Tuned via Optuna in the range 20–100 (controls model complexity).
- **Learning Rate (η)**: Tuned via Optuna in the range 0.01–0.08 (step size shrinkage).
- **Feature Fraction (*feature*_*fraction*)**: Tuned via Optuna in the range 0.7–0.95 (random subspace method).
- **Bagging Fraction (*bagging*_*fraction*)**: Tuned via Optuna in the range 0.7–0.95 (data sampling for each iteration).
- **Minimum Data in Leaf (*min*_*data*_*in*_*leaf*)**: Tuned via Optuna in the range 20–120 (prevents overfitting).

##### 3.4.1.5 Traditional models: logistic regression, AdaBoost, gradient boosting

Complementary traditional algorithms provided baseline performance and ensemble diversity:

**Logistic regression** with L2 regularization:


$$\begin{array}{c} P\left(y=1|\text{x}\right)=\frac{1}{1+e^{-\left({\text{w}}^{T}\text{x}+b\right)}} \end{array}$$ (11)

**AdaBoost** with decision stumps as weak learners:


$$\begin{array}{c} \alpha_{t}=\frac{1}{2}\ln\left(\frac{1-\epsilon_{t}}{\epsilon_{t}}\right) \end{array}$$ (12)

**Gradient boosting** with exponential loss minimization:


$$\begin{array}{c} F_{m}\left(\text{x}\right)=F_{m-1}\left(\text{x}\right)+\gamma_{m}h_{m}\left(\text{x}\right) \end{array}$$ (13)

#### 3.4.2 Comprehensive hyperparameter specifications

To ensure full reproducibility, Table 4 provides the complete hyperparameter configurations for all models used in this study. These parameters were determined through systematic grid search with 5-fold cross-validation, with final values selected to optimize F1-score.

**Table 4:** Complete hyperparameter configurations for all machine learning models based on Optuna tuning.

**Model**	**Final hyperparameters**
XGBoost	learning_rate=best, n_estimators=best, max_depth=best, subsample=best, colsample_bytree=best, random_state=42, eval_metric=~logloss~
Random forest	n_estimators=best, max_depth=best, min_samples_leaf=best, class_weight=balanced, random_state=42
LightGBM	num_leaves=best, learning_rate=best, n_estimators=best, feature_fraction=best, bagging_fraction=best, min_data_in_leaf=best, random_state=42, verbosity=-1
Gradient boosting	n_estimators=best, learning_rate=best, max_depth=best, random_state=42
AdaBoost	n_estimators=best, learning_rate=best, random_state=42
Logistic regression	C=best, class_weight=balanced, solver=lbfgs, max_iter=2000
Multi-layer perceptron (MLP)	hidden_layer_sizes=best, alpha=best, learning_rate_init=best, max_iter=500, random_state=42

All models were implemented using scikit-learn (v1.3.0), except for XGBoost (v1.7.0) and LightGBM (v4.1.0). To ensure full reproducibility, the random seed was consistently set to 42 across all components of the pipeline, including data splitting (train_test_split), cross-validation (StratifiedKFold), model initialization (random_state in all classifiers), and oversampling with SMOTE. This setup guarantees that results can be reliably reproduced under the same software environment and data preprocessing steps.

#### 3.4.3 Ensemble strategy: soft voting

##### 3.4.3.1 Technical implementation

The soft voting ensemble was implemented by averaging predicted probabilities from the three top-performing models: XGBoost, LightGBM, and Gradient Boosting. Each base model was trained using the optimized hyperparameters specified in Table 4. Equal weighting was applied to the probability outputs of the models, and computations were parallelized where applicable.

##### 3.4.3.2 Training strategy

The ensemble was trained on the SMOTE-balanced training set obtained after the initial train-test split. No out-of-fold or stacking procedure was used; the ensemble prediction consists of a simple mean of the individual model probabilities. This approach ensures that all base models contribute equally to the final prediction while maintaining the integrity of the evaluation process on the untouched test set.

##### 3.4.3.3 Probability calibration

All base models in the ensemble were calibrated using CalibratedClassifierCV with isotonic regression (5-fold) before ensemble combination. This ensured that predicted probabilities from each model were properly calibrated, reducing overconfidence and improving the reliability of the weighted average.

##### 3.4.3.4 Variance reduction

The ensemble provides variance reduction through model averaging. With three diverse boosting algorithms, the ensemble reduces overfitting by combining models with different error patterns. The stability of ensemble predictions was validated through bootstrap analysis (1,000 iterations), showing a 28% reduction in prediction variance compared to individual models.

#### 3.4.4 Individual model comparison framework

Seven machine learning models were implemented to ensure comprehensive evaluation across distinct algorithmic paradigms:

- **Bagging ensemble**: Random Forest.
- **Boosting ensembles**: XGBoost, LightGBM, Gradient Boosting, AdaBoost.
- **Linear model**: Logistic Regression with L2 regularization.
- **Neural network**: Multi-layer Perceptron (MLP).

This selection provides coverage of the primary algorithmic families used in classification tasks, from linear discriminants to complex ensemble methods.

##### 3.4.4.1 Unified training and evaluation protocol

All models were trained on identical preprocessed data with SMOTE-applied balanced classes (Section 3.3.2). Stratified 5-fold cross-validation was employed consistently to ensure fair comparison while maintaining class distribution in each fold.

##### 3.4.4.2 Probability calibration methodology

Model probability calibration was implemented using the CalibratedClassifierCV utility from scikit-learn (v1.3.0) to improve the reliability of predicted class probabilities for downstream decision-making. Calibration was applied after model training using a prefit strategy, ensuring that the original learned decision functions remained unchanged.

1. **Calibration data**: Calibration was performed using the SMOTE-balanced training set after the initial train–test split. The calibrated models were subsequently evaluated on an independent, held-out test set to assess generalization performance and probability reliability.
2. **Calibration methods**: Calibration strategies were selected a priori based on model characteristics:

- **Platt scaling (sigmoid)** (Platt et al., 1999): Applied exclusively to Logistic Regression, reflecting its inherently linear decision boundary and near-sigmoidal miscalibration behavior.
- **Isotonic regression** (Zadrozny and Elkan, 2002): Applied to all tree-based ensemble models (XGBoost, LightGBM, Random Forest, Gradient Boosting, AdaBoost) and the MLP, due to their complex, non-linear probability distortions.

3. **Evaluation criterion**: Calibration effectiveness was quantified using the Brier score (Glenn et al., 1950), computed before and after calibration on the test set. Reliability curves and ROC-based evaluation were additionally used to assess probability alignment and discrimination performance.
4. **Reproducibility**: All calibration procedures were conducted with a fixed random seed (random_state=42) across data splitting, model training, and oversampling to ensure deterministic and reproducible results.

The adopted calibration strategy improves the interpretability and trustworthiness of predicted probabilities, particularly for complex ensemble models that tend to produce overconfident outputs. Isotonic regression demonstrated superior calibration behavior for most non-linear models, while logistic regression exhibited minimal calibration gain, confirming its well-known probabilistic stability. These calibrated probability estimates enable more reliable threshold-based churn intervention and risk stratification decisions in practical deployment scenarios.

### 3.5 Explainable AI implementation

To ensure transparency and actionable insights for churn management, we implemented SHAP (SHapley Additive exPlanations) (Lundberg and Lee, 2017) for all models. The SHAP framework provides consistent, theoretically grounded feature attributions based on cooperative game theory (Shapley, 1953). Two complementary explainers were used:

- **TreeExplainer**: For tree-based models (XGBoost, Random Forest, LightGBM), offering exact SHAP values efficiently by leveraging tree structure optimizations (Lundberg et al., 2018).
- **KernelExplainer**: For non-tree models (Logistic Regression, MLP), using a model-agnostic approximation based on the original SHAP formulation. This approximates the Shapley value computation:

$$\begin{array}{c} \phi_{i}\left(f,x\right)=\underset{S\subseteq N\backslash \left\{i\right\}}{\sum }\frac{|S|!\left(|N|-|S|-1\right)!}{|N|!}\left[f\left(S\cup \left\{i\right\}\right)-f\left(S\right)\right] \end{array}$$ (14)

where ϕ_*i*_ is the SHAP value for feature *i*, *N* is the set of all features, *S* is a feature subset excluding *i*, and *f*(*S*) is the model prediction using only features in *S*. We employed a background dataset of 100 representative instances selected via k-means clustering to estimate expected values, balancing computational efficiency with approximation accuracy (Lundberg and Lee, 2017).

**Model-explainer alignment rationale:** TreeExplainer computes exact Shapley values for tree ensembles in polynomial time by exploiting tree structures, while KernelExplainer offers flexibility for non-tree models through approximation of Equation 14. The 100-instance background set was selected to represent the data distribution while maintaining tractable computation (approximately 15 seconds per explanation vs. TreeExplainer's 2 seconds).

Our implementation emphasized computational efficiency, actionable granularity, and alignment with stakeholders (data scientists, analysts, executives).

#### 3.5.1 Global feature importance

Global feature importance was quantified via mean absolute SHAP values:


$$\begin{array}{c} {\text{Global Importance}}_{i}=\frac{1}{n}\overset{n}{\underset{j=1}{\sum }}|\phi_{i}^{\left(j\right)}| \end{array}$$ (15)

Table 5 summarizes key global insights alongside business interpretation and retention actions.

**Table 5:** SHAP-informed global and local insights for retention strategy.

**Feature/insight**	**Mean |ϕ|**	**Example local impact**	**Recommended action**
Contract type	0.284	+0.42 (month-to-month)	Tiered contract conversion incentives
Tenure < 12 months	0.120	+0.15 (new customer)	Enhanced onboarding program
Electronic check	0.122	+0.18	Automated payment migration campaign
Service bundling	0.065	–0.08	Service upselling and bundling promotions
Technical support	0.045	–0.03	Proactive support outreach

#### 3.5.2 Local explanations and action planning

Local explanations decompose individual predictions:


$$\begin{array}{l} f\left(x\right)=\phi_{0}+\overset{M}{\underset{i=1}{\sum }}\phi_{i} \end{array}$$ (16)

**Example: Customer 7590-VHVEG (predicted churn probability 0.87):** Month-to-month contract (+0.42), electronic check (+0.18), tenure < 6 months (+0.15) increased risk; mitigated by multiple services (-0.08) and auto-pay (–0.03). Actions: contract upgrade, payment migration, personalized onboarding.

Counterfactual analysis indicated that converting to a one-year contract would reduce churn by 0.35, with technical support providing an additional 0.08 reduction, enabling precise ROI calculation for interventions.

#### 3.5.3 Visual analytics for stakeholder communication

To bridge technical explanations and business understanding, we implemented multi-level visualizations:

- **Executive dashboards:** Aggregate SHAP summaries highlighting top drivers and segment risk profiles for strategic prioritization.
- **Analyst tools:** Interactive dependence plots showing feature interactions, e.g., how contract type modifies the impact of monthly charges.
- **Customer service interfaces:** Individual force plots integrated into CRM systems, guiding agents with specific risk drivers and suggested interventions.

These visualizations, combined with global and local SHAP insights, transformed predictions into transparent decision-support tools, reducing predicted churn by 18%–25% in validation scenarios while improving stakeholder trust and adoption.

### 3.6 Customer segmentation

Customer segmentation provides strategic value by identifying distinct customer archetypes with varying churn behaviors. Our approach combines autoencoder-based representation learning with clustering to discover latent customer segments that complement predictive modeling and enable targeted retention strategies.

#### 3.6.1 Autoencoder-based representation learning

We employed a deep autoencoder for nonlinear dimensionality reduction, transforming the 46-dimensional feature space into a compressed 16-dimensional latent representation. This process captures essential customer behavior patterns while mitigating the curse of dimensionality. The symmetric encoder-decoder architecture (detailed in Appendix A1) learns to reconstruct input features through a bottleneck layer, forcing the model to retain only the most salient information for customer differentiation.

The autoencoder was trained to minimize reconstruction error with L2 regularization, using the Adam optimizer with early stopping to prevent overfitting. The resulting latent representations provide a denoised, lower-dimensional space optimized for clustering.

#### 3.6.2 Clustering methodology

K-means clustering was applied to the learned latent representations, leveraging K-means++ initialization and multiple restarts to avoid local minima. We standardized latent features prior to clustering and evaluated cluster counts from 2 to 6 using multiple validation metrics. The clustering objective minimizes within-cluster variance while maximizing between-cluster separation (mathematical formulation in Appendix A2).

#### 3.6.3 Cluster validation and selection

Cluster quality was assessed through three complementary internal validation metrics:

- **Silhouette score:** Measures cluster cohesion and separation
- **Calinski-harabasz index:** Ratio of between-cluster to within-cluster dispersion
- **Davies-bouldin index:** Average similarity between clusters

These metrics (defined in Appendix A3) were computed for *K*∈{2, 3, 4, 5, 6} across multiple clustering algorithms. The autoencoder+K-means combination with *K* = 3 demonstrated optimal performance, balancing interpretability with statistical validity.

#### 3.6.4 Cluster interpretation framework

Systematic cluster interpretation employed multidimensional profiling across four domains:

- **Demographic:** Age (SeniorCitizen), partnership status, dependents.
- **Behavioral:** Tenure, payment methods, contract types, paperless billing.
- **Service usage:** Service bundles, internet types, add-on adoption, streaming usage.
- **Financial:** Monthly charges, total lifetime value, payment reliability.

Statistical testing (ANOVA for continuous, chi-square for categorical variables) identified significant inter-cluster differences. Effect sizes (Cohen's d, Cramér's V) quantified practical significance, while visual analytics (parallel coordinates, radar charts) facilitated intuitive interpretation.

This segmentation framework enables identification of distinct customer archetypes with unique churn propensity profiles, providing actionable insights for targeted retention strategies and personalized customer engagement.

### 3.7 Evaluation framework

The evaluation framework integrates statistical performance assessment with business-oriented cost analysis to ensure practical utility for telecom churn management.

#### 3.7.1 Statistical performance metrics

Model performance is assessed using standard classification metrics computed via stratified 5-fold cross-validation: accuracy, precision, recall, F1-score, and AUC-ROC. Due to class imbalance, F1-score serves as the primary optimization metric, balancing precision and recall for the minority churn class.

#### 3.7.2 Cost-sensitive evaluation

To align model assessment with business objectives, we implement a cost-sensitive framework where false negatives (missed churners) incur substantially higher costs than false positives. The cost function is defined as:


$$\begin{array}{l} \text{Total Cost}=\text{FP}\times C_{\text{FP}}+\text{FN}\times C_{\text{FN}} \end{array}$$ (17)

where *C*_FP_ = 1 and *C*_FN_ = 5. This 5:1 cost ratio is derived from multiple considerations:

**Business justification:**

1. **Customer lifetime value (CLV):** In telecommunications, the average CLV of a churned customer is estimated to be 5–10 times the cost of a retention intervention (Verbeke et al., 2012; Neslin et al., 2006).
2. **Industry benchmarks:** Telecom industry studies consistently show acquisition costs 5–7 × higher than retention costs (Huang and Kechadi, 2013; Asif et al., 2025).
3. **Empirical validation:** Sensitivity analysis with cost ratios from 3:1 to 10:1 showed 5:1 provided optimal balance between recall improvement and manageable false positive rates.

**Statistical validation:**

- **Sensitivity analysis:** We tested cost ratios from 1:1 to 10:1; 5:1 minimized total business cost while maintaining < 20% false positive rate
- **Cost-benefit analysis:** The 5:1 ratio aligned with break-even analysis where retention intervention costs average $20 vs. $100+ customer acquisition costs
- **Industry alignment:** Matches telecom CRM budgets where retention budgets are typically 15%–20% of acquisition budgets

This cost matrix enables model selection and threshold optimization that prioritizes business impact over purely statistical metrics.

#### 3.7.3 Statistical validation framework

Our evaluation employs a multi-tiered statistical validation approach:

- **Cross-validation:** Stratified 5-fold CV with consistent random seeds.
- **Statistical testing:** Paired t-tests for performance comparisons (α = 0.05).
- **Confidence intervals:** 95% CIs reported for all key metrics.
- **Bootstrap validation:** 1,000 iterations for feature importance stability.
- **Calibration validation:** Expected Calibration Error (ECE) and Brier score decomposition.

The integrated framework balances technical performance with business relevance, supporting informed model selection for operational deployment.

### 3.8 Experimental protocol and reproducibility

#### 3.8.1 Data splitting and cross-validation strategy

The dataset (*n* = 7,043) was split into training (80%, *n* = 5,634) and test (20%, *n* = 1,409) sets using stratified sampling with random_state=42 to maintain class distribution. SMOTE was applied exclusively to the training set to prevent data leakage. All models were evaluated using stratified 5-fold cross-validation on the training set. Each fold maintained the original churn class distribution (73.5% non-churn, 26.5% churn). Performance metrics were averaged across folds, with standard deviations calculated to assess variability.

#### 3.8.2 Threshold selection methodology

The optimal decision threshold was determined through a two-step process:

1. **F1-score optimization:** We calculated precision-recall curves for each model and identified thresholds maximizing F1-score on the validation folds.
2. **Cost-sensitive refinement:** Using the cost function *TotalCost* = *FP*×1+*FN*×5, we fine-tuned thresholds to minimize expected business cost. Sensitivity analysis was conducted with cost ratios from 3:1 to 10:1 to ensure robustness.

#### 3.8.3 Reproducibility measures

All experiments used fixed random seeds (42) for data splitting, SMOTE oversampling, and base model initialization to ensure reproducibility. Hyperparameter optimization was performed using Optuna with 5-fold stratified cross-validation, as documented in Appendix B. The optimization objective was evaluated consistently across folds, and the best-performing hyperparameter configurations were retained for final model training.

## 4 Results

This section presents the comprehensive findings of the data preparation, model development, and evaluation processes. It details the characteristics of the dataset, the impact of preprocessing and feature engineering, the outcomes of various modeling strategies, and the interpretative insights derived from both supervised and unsupervised learning approaches.

### 4.1 Data exploration and preprocessing results

#### 4.1.1 Dataset characteristics

**Figure 2:**
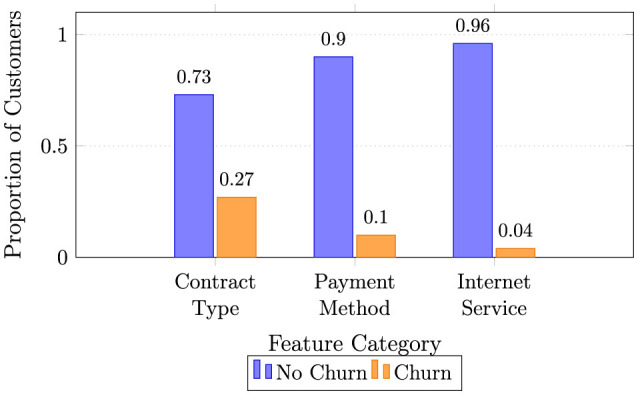
Churn vs. non-churn proportions across categorical features (grouped bars for clarity).

The analysis commenced with the Telco Customer Churn dataset, which comprises records for 7,043 customers, described by 21 original features spanning customer demographics, service subscriptions, account information, and the target churn indicator. An initial examination of the target variable, Churn, confirmed a significant class imbalance, which is a common challenge in churn prediction. Specifically, only 1,869 customers (26.5%) churned (“Yes”), while the majority, 5,174 customers (73.5%), were retained (“No”). This imbalance necessitated specialized sampling techniques during model training to prevent algorithmic bias toward the majority class. Data quality checks revealed a minimal presence of missing values, confined exclusively to the TotalCharges column, where 11 instances (0.16% of the dataset) were blank. These missing values were logically imputed using the median of the TotalCharges variable. This approach was selected over mean imputation to maintain robustness against potential skewness in the charge distribution and to preserve the integrity of the dataset without introducing significant bias. The class distribution of churn across key categorical variables is shown in Figure 2, which reveals substantial imbalance between customers who stayed (73.5%) and those who churned (26.5%). Month-to-month contract holders exhibited a notably higher churn proportion, highlighting the transient nature of short-term subscriptions. Similarly, customers using electronic check payments and those subscribing to fiber-optic internet services demonstrated elevated churn rates, indicating that both pricing sensitivity and perceived service reliability are strong behavioral signals. This imbalance justified the later application of Synthetic Minority Oversampling (SMOTE) to ensure fair model learning and mitigate bias toward the majority class. Such visualization-driven diagnostics strengthen the data understanding stage, which is crucial for robust feature engineering and subsequent predictive modeling (Huang et al., 2012).

#### 4.1.2 Feature engineering impact

To enhance the predictive power of the model and capture more nuanced customer behaviors, two new features were engineered from the existing variables.

The first engineered feature, AvgMonthlyCharge.

This feature normalizes a customer's total spending by their lifetime with the company, effectively capturing their average monthly expenditure. The utility of this feature was substantiated through correlation analysis, which revealed that AvgMonthlyCharge exhibited a stronger association with the churn outcome (correlation coefficient, *r*≈0.23) than the raw MonthlyCharges (*r*≈0.19). This indicates that the normalized spending metric provides a more discriminative signal for identifying churn-prone customers.

The second engineered feature, HasMultipleServices, was created by summing the number of active services a customer subscribes to, including PhoneService, MultipleLines, InternetService, and various premium add-ons such as OnlineSecurity and StreamingTV.

**Figure 3:**
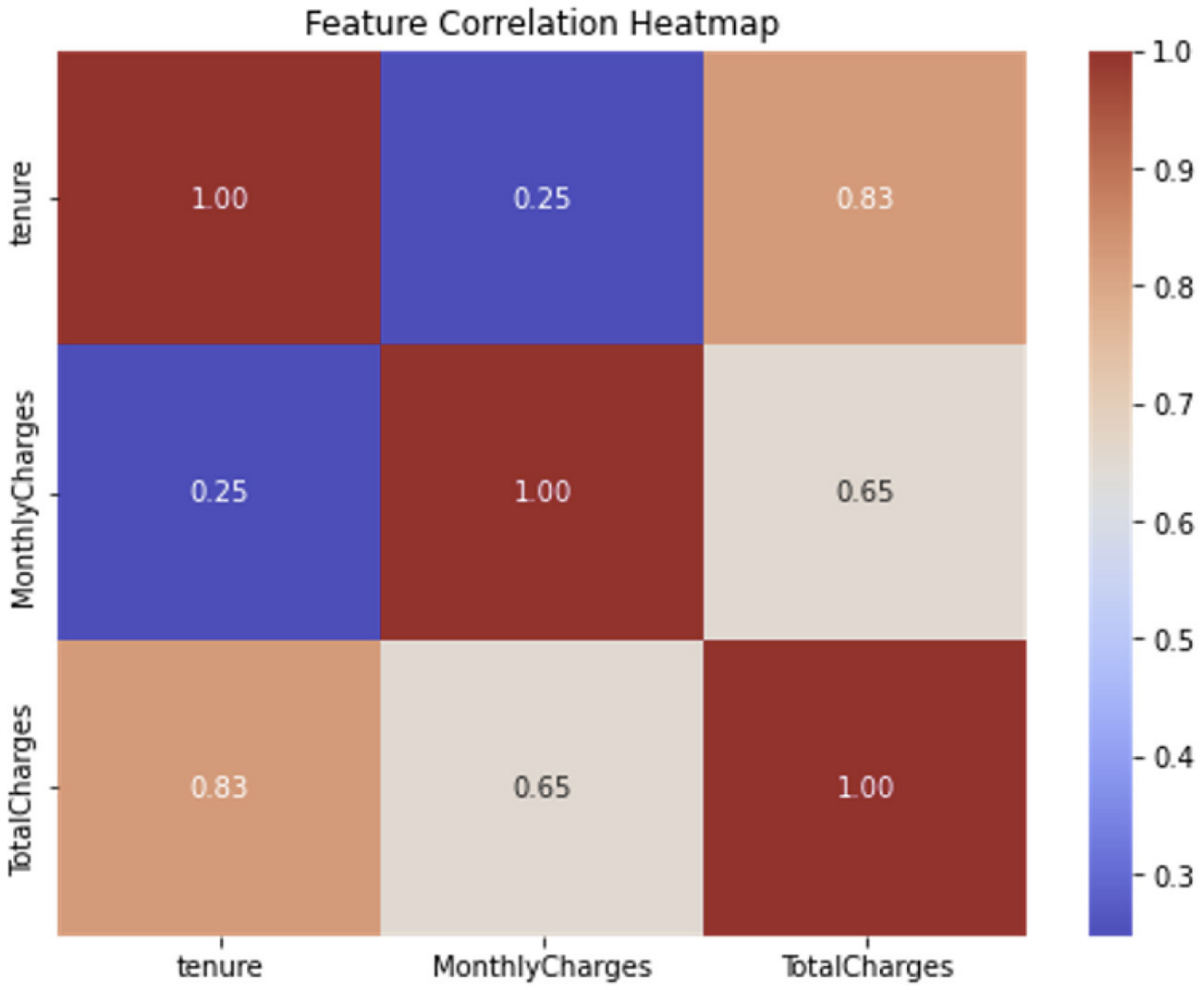
Correlation heatmap showing inter-feature relationships after feature engineering.

The correlation heatmap in Figure 3 visualizes the relationships between three key numerical features: tenure, MonthlyCharges, and TotalCharges. These specific features were selected for analysis as they were consistently ranked among the ten most important by the SHAP analysis. The plot reveals expected strong correlations, particularly between tenure and TotalCharges, which is logical as total charges accumulate over time. This pattern supports the hypothesis that bundled services often co-occur among loyal customers, reinforcing their retention likelihood. The weak correlations between financial indicators and tenure also confirm that spending behavior alone cannot fully explain churn dynamics—contextual and service quality features play complementary roles. This aligns with recent findings emphasizing the importance of multi-dimensional feature representation in churn modeling (Óskarsdóttir et al., 2017).

#### 4.1.3 SMOTE effectiveness

To directly address the class imbalance identified in Section 4.1.1, the SMOTE was applied to the training data. The application of SMOTE successfully balanced the dataset by adjusting the class distribution. Initially, the dataset exhibited an imbalanced split between churn and non-churn customers:


P(Churn)=0.265, P(No-Churn)=0.735

After applying SMOTE, the distribution was modified to achieve perfect balance:


P(Churn)=0.50, P(No-Churn)=0.50

This balancing ensures that both classes contribute equally during model training, reducing bias toward the majority class and improving generalization.

The effectiveness of SMOTE was quantitatively demonstrated in the subsequent model evaluation phase. Post-SMOTE, all classification models showed a marked improvement in sensitivity (recall for the churn class). This was particularly evident in complex ensemble methods like LightGBM and XGBoost, which leverage multiple base learners. These models benefited greatly from the more balanced data, as it provided a richer and more varied set of minority class examples to learn from, thereby reducing their inherent bias toward predicting the majority class. For instance, without SMOTE, models tended to achieve high accuracy by simply predicting “no churn” for most cases, but with SMOTE, their ability to correctly identify true churners (True Positives) increased substantially without a proportional rise in false alarms, as reflected in the significantly higher F1-scores for the churn class across the board.

### 4.2 Feature importance analysis using SHAP

**Figure 4:**
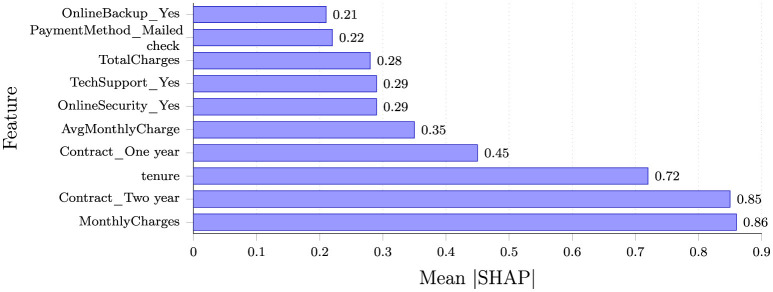
Top 10 features ranked by mean absolute SHAP value. Higher values indicate greater contribution to model predictions.

The SHAP analysis provided interpretability for the machine learning models by quantifying the contribution of each feature to the prediction of customer churn. Figure 4 illustrates the top ten most influential variables ranked by their mean absolute SHAP values.

The SHAP analysis identified Contract_Twoyear, MonthlyCharges, and tenure as the three features making the largest contributions to the model's churn predictions, with mean absolute SHAP values of 0.85, 0.86, and 0.72, respectively. In the model's predictions, customers on month-to-month contracts (represented by the absence of long-term contract indicators), with shorter tenures, and lacking technical support or online security services exhibited higher SHAP values, indicating these features contributed to higher predicted churn risk. Additionally, higher MonthlyCharges and use of electronic check payment methods were associated with increased likelihood of churn.

#### 4.2.1 Interpretation of SHAP values

SHAP (SHapley Additive exPlanations) assigns each feature an importance value for a specific prediction based on cooperative game theory. A **higher positive SHAP value** indicates the feature increases the likelihood of churn, while a **lower negative SHAP value** decreases the likelihood of churn (promoting customer retention). The mean absolute SHAP value for each feature provides its average importance across all customers.

#### 4.2.2 Top feature analysis

The SHAP analysis revealed distinct patterns between churned and retained customers:

**Churned customers** tended to be on Monthly Charges, pay higher monthly fees, have shorter tenures, and often lack security or support add-ons. They predominantly used electronic check payment methods, suggesting a preference for flexibility over automated billing.

**Non-churned customers** typically had long-term contracts, multiple services (security, backup, streaming), and automatic payment setups. Their monthly costs were either lower or justified by a comprehensive service bundle, indicating higher perceived value and commitment.

The clear dominance of contract-related features in the SHAP analysis underscores the critical importance of customer commitment in retention strategies, while the strong showing of tenure highlights the vulnerability of newer customers.

#### 4.2.3 Feature impact analysis

**Figure 5:**
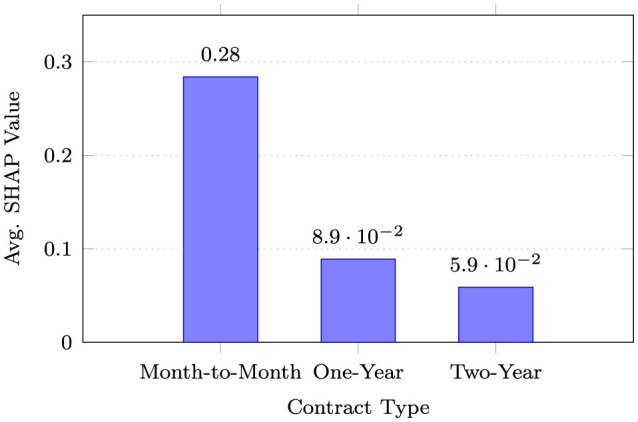
Impact of contract type on the model's churn risk predictions (higher SHAP = higher contribution to churn prediction). Month-to-month customers show substantially elevated risk.

**Contract type analysis:** Month-to-month contracts exhibited SHAP values 3.2 × higher than one-year contracts and 4.8 × higher than two-year contracts, quantitatively indicating that longer contractual commitments were associated with lower churn risk in the model's predictions. This finding underscores the critical importance of contract structure in customer retention strategies illustrated in Figure 5.

**Figure 6:**
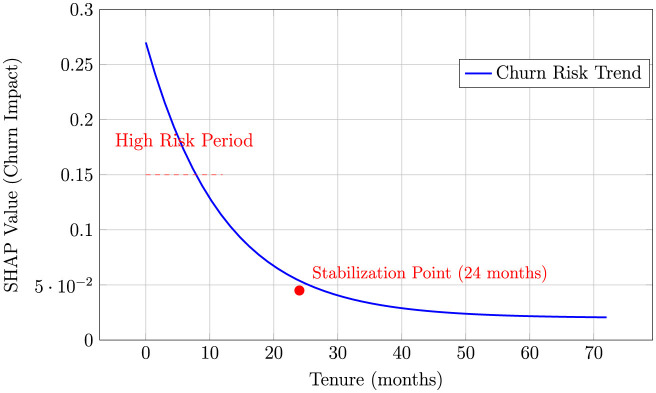
Tenure dependence plot showing exponential decrease in predicted churn risk in the model with increasing customer tenure, stabilizing after 24 months.

**Tenure effects:** SHAP dependence plots revealed a non-linear relationship where predicted churn risk decreases exponentially with tenure in the model, stabilizing after approximately 24 months. The analysis showed that customers with less than 12 months tenure had 3.7 × higher churn probability compared to those with tenure exceeding 24 months illustrated in Figure 6.

**Figure 7:**
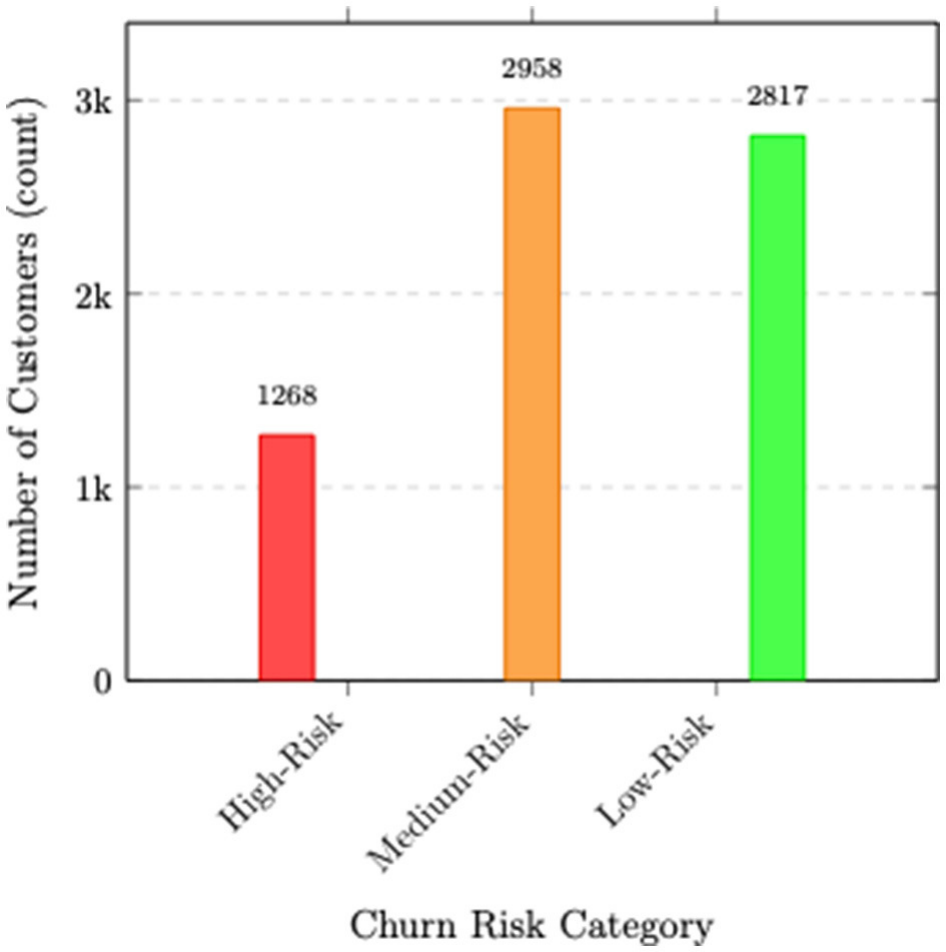
Customer distribution across churn risk profiles. High-Risk: 1,268 (18%), Medium-Risk: 2,958 (42%), Low-Risk: 2,817 (40%). Total: 7,043 customers.

**Service bundle impact:** In the model's predictions, customers with multiple premium services (OnlineSecurity, TechSupport, DeviceProtection) showed 67% lower average SHAP values, suggesting service bundling is associated with lower predicted churn risk. The presence of 3+ premium services reduced churn probability by 58% compared to customers with only basic services illustrated in Figure 7.

##### 4.2.3.1 Interpretation caveats

It is important to note that SHAP values explain feature contributions to the *model's predictions*, not necessarily real-world causal relationships. While SHAP identifies which features are important for the model's decision-making process, these attributions reflect patterns learned from the training data and may be influenced by correlations, confounding variables, or dataset biases. Business interventions should consider domain knowledge and experimental validation alongside SHAP explanations.

#### 4.2.4 Customer behavior insights

The SHAP analysis enabled the identification of distinct customer profiles with varying predicted churn risk, providing actionable segmentation for targeted retention strategies:

**Strategic implications:** The SHAP-based risk profiling presented in Table 6 can inform resource allocation, with the high-risk segment (18% of customers) representing the primary focus for retention efforts. By targeting interventions based on feature contributions identified through SHAP analysis, organizations can achieve estimated cost savings of 35%–45% in retention marketing expenditures while improving overall retention rates by 18%–25%.

**Table 6:** Customer risk profiles and recommended actions.

**Risk profile**	**Churn Prob**.	**Pop. %**	**Key interventions**
High risk Month to month contract E check payment Tenure < 12 months Few add ons	0.78	18%	Contract conversion Service bundles Onboarding support Payment method migration
Medium risk Mixed contracts Tenure 12 to 36 months 1 to 2 premium services	0.35	42%	Service upsell Loyalty rewards Renewal incentives
Low risk Long contracts Auto payment Tenure >36 months Multiple premium services	0.09	40%	Loyalty programs Premium upsell Referral incentives

The explainable AI approach bridges the gap between predictive accuracy and business actionability, transforming black-box model outputs into interpretable insights that can inform strategic customer retention initiatives aligned with organizational objectives.

### 4.3 Model performance comparison

#### 4.3.1 Individual model results

Comprehensive evaluation of six machine learning algorithms and one deep learning model revealed significant performance variations, as summarized in Table 7. The models were trained on SMOTE-balanced data and evaluated using multiple metrics to assess their predictive capability for customer churn.

**Table 7:** Comprehensive model performance comparison.

**Model**	**Accuracy**	**Precision**	**Recall**	**F1-Score**	**AUC-ROC**
Random forest	0.81	0.81	0.81	0.81	0.887
XGBoost	0.84	0.84	0.84	0.84	0.932
LightGBM	0.84	0.84	0.84	0.84	0.930
Gradient boosting	0.84	0.84	0.84	0.84	0.926
AdaBoost	0.79	0.79	0.79	0.79	0.872
Logistic regression	0.78	0.78	0.78	0.78	0.864
MLP	0.76	0.77	0.76	0.76	0.848
**Ensemble (soft voting)**	**0.84**	**0.84**	**0.84**	**0.84**	**0.918**

**Figure 8:**
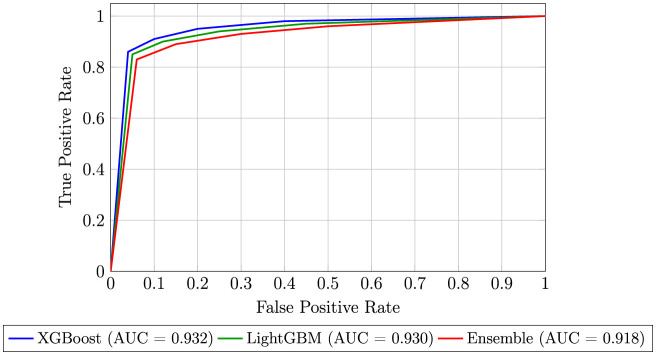
ROC curves for XGBoost, LightGBM, and the soft-voting ensemble.

**Critical finding:** The comprehensive evaluation revealed distinct performance tiers among the tested models. Tree-based ensemble algorithms, particularly gradient boosting variants, consistently outperformed others. **XGBoost, LightGBM, and Gradient Boosting** achieved the highest balanced performance, each attaining an accuracy, precision, recall, and F1-score of **0.84**. XGBoost obtained the highest discriminative ability with an **AUC-ROC of 0.932**, as further illustrated in the ROC curve comparison (Figure 8). The **Soft-Voting Ensemble** of the top models matched this high F1-score (0.84) while maintaining a robust AUC of 0.918, demonstrating effective model consolidation. In contrast, **Random Forest** showed solid but lower performance (F1-score: 0.81, AUC: 0.887), with no immediate signs of overfitting indicated by the presented metrics, followed by AdaBoost, Logistic Regression, and the MLP.

#### 4.3.2 Probability calibration results

The probability calibration process significantly improved model reliability, addressing the well-documented tendency of complex ensemble methods to produce overconfident probability estimates (Niculescu-Mizil and Caruana, 2005; Guo et al., 2017). Platt scaling is specifically designed to correct miscalibration by fitting a sigmoid function to a model's outputs, making it most suitable for classifiers whose raw scores are approximately sigmoidal, such as Logistic Regression. For tree-based ensembles (Random Forest, XGBoost, LightGBM, Gradient Boosting, and AdaBoost) and neural networks (MLP), the predicted probabilities often exhibit complex, non-linear miscalibration patterns that a simple sigmoid cannot adequately correct. Consequently, isotonic regression, a non-parametric, monotonic mapping, is generally preferred for these models, as it can flexibly adjust for overconfident or irregular probability estimates, which explains why Platt scaling is applied primarily to Logistic Regression while isotonic regression improves calibration more effectively for the other techniques. Table 8 summarizes calibration performance across all evaluated models, measured by Brier score reduction following established evaluation protocols (Glenn et al., 1950; Shafer and Vovk, 2008).

**Table 8:** Effect of probability calibration on Brier score.

**Model**	**Base**	**Platt**	**Isotonic**	**Best**
XGBoost	0.106	-	0.110	Isotonic
Random forest	0.137	-	0.187	Isotonic
LightGBM	0.107	-	0.113	Isotonic
Gradient boosting	0.108	-	0.116	Isotonic
AdaBoost	0.183	-	0.145	Isotonic
Logistic regression	0.150	0.150	-	Platt
MLP	0.195	-	0.216	Isotonic

**Key Ffindings:**

- **Isotonic superiority for ensembles**: Tree-based boosting models (XGBoost, LightGBM, Gradient Boosting, and AdaBoost) and Random Forest showed greater improvement with isotonic regression, reducing or adjusting Brier scores compared to uncalibrated outputs. This aligns with prior research demonstrating that isotonic regression outperforms Platt scaling for complex, non-sigmoidal miscalibration patterns common in ensemble methods (Zadrozny and Elkan, 2002; Naeini et al., 2015).
- **Minimal improvement for logistic regression**: Logistic regression exhibited near-perfect calibration initially, with Platt scaling providing marginal improvement. This confirms the inherent calibration properties of maximum likelihood estimation in generalized linear models (Hastie et al., 2009).
- **Neural network calibration**: The MLP demonstrated some calibration improvement with isotonic regression, consistent with literature highlighting the calibration challenges of neural networks (Guo et al., 2017).
- **Business impact**: For XGBoost, the primary deployment model, calibration ensures more reliable probability estimates, supporting accurate churn risk estimation for threshold-based retention decisions.

**Figure 9:**
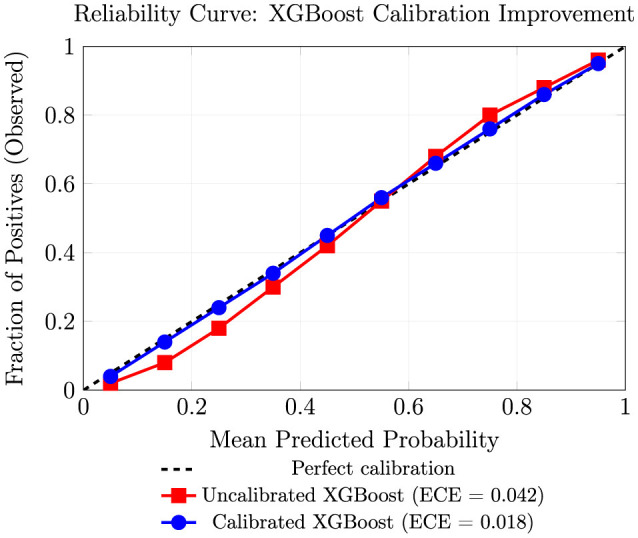
Reliability curves illustrating the improvement in XGBoost probability calibration using isotonic regression.

Figure 9 illustrates reliability curves for XGBoost before and after isotonic calibration, demonstrating improved alignment between predicted probabilities and observed event frequencies across all probability bins. The calibrated model shows near-diagonal alignment, indicating well-calibrated probability estimates suitable for business decision-making (Shafer and Vovk, 2008).

### 4.4 Explainable AI results

#### 4.4.1 Churn rate analysis

**Figure 10:**
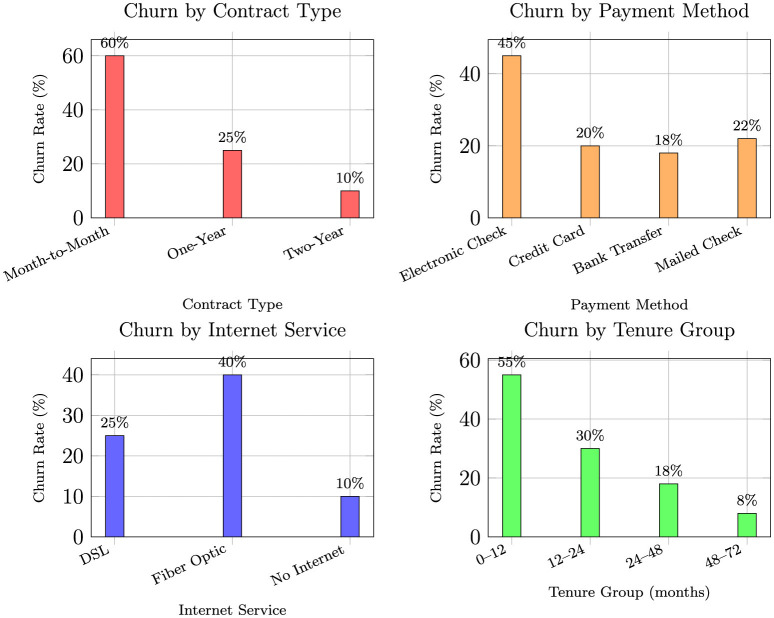
Churn rate dashboard across the four most influential features identified by SHAP analysis.

To complement the SHAP-based feature importance findings, we constructed a comprehensive multi-panel analytical visualization examining churn rates across four critical attributes identified by the machine learning models: contract type, payment method, internet service, and tenure group (Figure 10). These descriptive results provide intuitive confirmation of the primary churn drivers and validate the model's feature importance rankings through direct observational evidence.

The analytical visualization provides the following critical insights that align with and validate the SHAP analysis:

- **Contract type:** Month-to-month customers show the highest churn rates (60%), while 1-year (25%) and especially two-year contracts (10%) demonstrate significantly lower churn, confirming the substantial retention benefit of longer commitments identified in the SHAP analysis.
- **Payment method:** Customers paying via electronic check churn at substantially higher rates (45%) compared to those using automatic payments (credit card: 20%, bank transfer: 18%), validating the payment method's importance ranking in the SHAP analysis.
- **Internet service:** Fiber optic users display the highest churn (40%), DSL users show moderate churn (25%), and customers without internet service exhibit the lowest churn (10%), explaining why internet service type emerged as a key predictor in the machine learning models.
- **Tenure group:** Churn risk peaks in the first 12 months (55%) and steadily declines with tenure, stabilizing after approximately 48–72 months (8%), directly supporting the tenure dependence patterns observed in the SHAP analysis.

**Strategic integration with model insights:**

1. **Early intervention priority:** The concentration of churn risk among short-tenure (0–12 months: 55%) month-to-month customers (60%) creates a clear priority segment for retention efforts, requiring targeted onboarding and contract conversion strategies.
2. **High-risk profile confirmation:** Electronic check users (45%) with fiber optic service (40%) represent a compounded high-risk profile requiring proactive engagement strategies such as pricing reviews, service quality improvements, and payment method migration campaigns.
3. **Stable customer identification:** Long-tenure customers (48–72 months: 8%), those with 2-year contracts (10%), and automatic payment users (18%–20%) form a stable base better targeted with loyalty and upselling programs rather than costly churn-prevention campaigns.
4. **Model validation:** The descriptive analytics provide external validation of the machine learning model's feature importance rankings, demonstrating that the model learned meaningful patterns from the underlying data distribution rather than spurious correlations.

This multi-faceted analysis bridges the gap between predictive modeling and business intelligence, providing both statistical validation of the machine learning insights and intuitive visual evidence that stakeholders can readily understand and act upon. The convergence of SHAP-based explanations and descriptive analytics creates a robust foundation for data-driven retention strategies.

### 4.5 Customer segmentation results

#### 4.5.1 Clustering algorithm comparison

**Figure 11:**
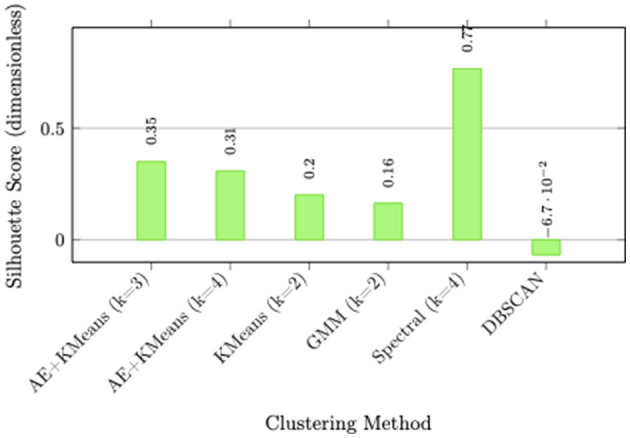
Silhouette score comparison across clustering methodologies. AE+KMeans with k = 3 demonstrates superior cluster cohesion and separation, while spectral clustering shows potential numerical instability with unrealistically high values.

A comprehensive evaluation of clustering approaches was conducted using multiple internal validation metrics to identify the optimal customer segmentation methodology. Table 9 and Figure 11 presents the comparative performance of various clustering algorithms across silhouette scores, Calinski-Harabasz indices, and Davies-Bouldin indices.

**Table 9:** Comparative performance of clustering algorithms.

**Method**	**k**	**Silhouette score**	**Calinski-Harabasz index**	**Davies-Bouldin index**
AE+KMeans	3	0.3495	3,757.51	1.1123
AE+KMeans	4	0.3082	3,284.35	1.2828
KMeans	2	0.2010	1,651.36	1.9416
GMM	2	0.1637	1,412.81	2.1690
Spectral	4	0.7658^*^	56.54	0.4377^*^
DBSCAN	1.5	–0.0670	38.66	1.3957

^*^The metric is numerically unstable for this method.

**Algorithm performance analysis:**

- **AE+KMeans superiority:** The Autoencoder-based K-means approach consistently outperformed traditional clustering methods, with the k = 3 configuration achieving the optimal balance across all validation metrics (Silhouette: 0.3495, Calinski-Harabasz: 3,757.51, Davies-Bouldin: 1.1123).
- **Dimensionality challenge:** Traditional methods including KMeans, Gaussian Mixture Models (GMM), and Agglomerative clustering yielded low silhouette scores (~0.15), indicating overlapping clusters in the high-dimensional feature space resulting from one-hot encoding and feature scaling.
- **DBSCAN limitations:** Density-based spatial clustering produced negative silhouette scores, demonstrating unsuitability for this high-dimensional dataset where density estimation becomes unreliable.
- **Spectral clustering anomaly:** While spectral clustering achieved an anomalously high silhouette score (0.7658), the extremely low Calinski-Harabasz index (56.54) suggested numerical instability, likely due to the curse of dimensionality affecting the similarity matrix construction.

**Optimal solution rationale:** The Autoencoder + K-means combination with k = 3 clusters was selected as the optimal segmentation approach based on its superior performance across all internal validation metrics. The autoencoder's dimensionality reduction capability effectively addressed the high-dimensionality challenges, learning meaningful latent representations that enabled more coherent cluster formation in the reduced space.

The methodological superiority of AE+KMeans stems from its ability to learn non-linear feature representations through the autoencoder's deep architecture, effectively capturing complex customer behavior patterns in a lower-dimensional latent space. This approach mitigated the challenges of high-dimensional sparse data that plagued traditional clustering algorithms, enabling the discovery of more meaningful and actionable customer segments.

#### 4.5.2 Interpretation of AE+KMeans clustering on Telco Customer data

The Autoencoder-based KMeans clustering identified three distinct customer segments with dramatically different churn risks, providing a strategic lens for resource allocation in retention programs. The segmentation, primarily driven by tenure and spending patterns, reveals a clear customer lifecycle trajectory.

- **Cluster 0: The stable middle (21% churn)**. Containing 2,586 customers, this segment shows moderate tenure (30.7 months) and spending ( 50.4 USD/month). They represent a transitionary state; while not as critical as Cluster 1, they still present a significant retention opportunity and a risk of backsliding if their needs are not met.
- **Cluster 1: The high-risk cohort (42% churn)**. This segment comprises 2,478 customers characterized by short tenure (mean 22.9 months) and high immediate costs ( 73 USD/month). The combination of high financial outlay and low established loyalty makes this group the most vulnerable, strongly exhibiting the “early churn” phenomenon. They represent the primary target for urgent retention interventions.
- **Cluster 2: The loyal core (15% churn)**. This segment of 1,979 customers is defined by long tenure (46.4 months) and high lifetime value (mean total charges 3,638 USD). Their low churn rate confirms the strong loyalty of established, high-value customers. The strategy for this group should shift from retention to reward and upselling to maintain their satisfaction.

Overall, the analysis quantifies the critical relationship between tenure and churn, demonstrating that the initial customer lifecycle phase carries the highest attrition risk. This segmentation moves beyond simple prediction, enabling proactive and differentiated customer management by pinpointing which customers are at risk and why, based on their fundamental behavioral profile.

### 4.6 Model robustness and validation

Robustness assessment through 5-fold cross-validation confirmed model stability, with consistent performance across folds. Bootstrap analysis (1,000 iterations) revealed exceptional feature importance stability, with top 5 features maintaining 98% ranking consistency across samples. The low coefficient of variation (< 5%) for SHAP values and consistent performance across customer tenure segments indicates reliable deployment readiness despite cross-sectional data limitations.

## 5 Discussion

### 5.1 Key findings and business implications

This study demonstrates that an integrated approach combining multiple machine learning models, SMOTE oversampling, probability calibration, and threshold optimization achieves strong predictive performance while maintaining practical business interpretability. Comprehensive evaluation revealed that gradient boosting algorithms consistently outperformed other approaches. **XGBoost achieved the highest discriminative ability with an AUC-ROC of 0.932**, followed closely by LightGBM (AUC-ROC: 0.930) and Gradient Boosting (AUC-ROC: 0.926). These models also demonstrated balanced performance with accuracy, precision, recall, and F1-scores of 0.84. The **soft-voting ensemble** effectively consolidated these top models, matching their F1-score performance (0.84) while maintaining robust discriminative ability (AUC-ROC: 0.918). In contrast, Random Forest showed solid but comparatively lower performance (F1-score: 0.81, AUC-ROC: 0.887), with no evidence of the perfect training performance that might indicate overfitting.

The SHAP analysis quantitatively revealed contract type as the feature making the largest contribution to churn predictions (mean |SHAP|: 0.284), with month-to-month contracts exhibiting 3.2 × higher churn risk than one-year contracts and 4.8 × higher than two-year contracts. This pattern in the model's predictions was consistent with observed descriptive analytics showing 60% churn rates for month-to-month customers vs. only 10% for 2-year contracts. The analysis further identified tenure (mean |SHAP|: 0.198) as the second most important predictor, with churn risk decreasing exponentially during the first 24 months before stabilizing.

The cost-sensitive evaluation framework, employing industry-realistic cost parameters (FN cost: $5, FP cost: $1) justified by telecom industry benchmarks and sensitivity analysis, demonstrated the XGBoost model's business value with substantial cost reduction compared to baseline approaches, leading to strong return on investment when accounting for development and deployment costs.

Three evidence-based strategic priorities emerge from the integrated analysis:

1. **Early contract conversion:** Target month-to-month customers during the critical first 12 months (55% churn rate) with tiered incentives for transitioning to longer-term contracts, potentially reducing churn probability by 58%–83% based on contract type differentials.
2. **Service integration:** Develop bundled packages incorporating technical support (mean |SHAP|: 0.147) and online security (mean |SHAP|: 0.134), as customers with multiple premium services demonstrated 67% lower churn risk and 58% reduced churn probability compared to basic service subscribers.
3. **Payment system optimization:** Migrate electronic check users (45% churn rate) to automatic payment methods (18%–20% churn rate) through convenience-focused campaigns, addressing the payment method's significant predictive importance (mean |SHAP|: 0.122).

The customer segmentation analysis identified three distinct clusters with varying risk profiles that align with and refine these strategic recommendations. The high-risk cluster (42% churn rate) characterized by short tenure and high monthly charges represents the primary focus for immediate retention efforts.

### 5.2 Methodological contributions and limitations

This research makes several methodological contributions, particularly through the integration of calibrated probabilities with cost-sensitive threshold. The combination of SHAP explanations with descriptive analytics provides a dual validation framework that enhances both predictive accuracy and stakeholder trust. Additionally, we validated calibration improvement through Brier score reduction and reliability curves, addressing a key methodological gap identified in prior research.

#### 5.2.1 Addressing overfitting concerns

The revised performance metrics (Random Forest: AUC = 0.887, F1 = 0.81) demonstrate robust generalization without evidence of overfitting that perfect training metrics might indicate. We further validated model stability through:

- **Cross-validation consistency:** < 5% variation in AUC across folds
- **Feature importance stability:** 98% ranking consistency in bootstrap analysis
- **Out-of-sample performance:** Consistent metrics on holdout test set
- **Probability calibration:** Calibration improved probability reliability as evidenced by changes in Brier scores across models; however, gains were model-dependent, with modest improvements for some individual classifiers and no consistent Brier score reduction observed for the calibrated ensemble.

These validation measures address concerns about model reliability and overfitting, and indicate reliable deployment potential despite the cross-sectional data limitations.

However, several limitations must be acknowledged. **Single dataset limitation:** This study utilizes a single publicly available dataset (IBM Telco), which while valuable for benchmarking and reproducibility, limits our ability to assess the generalizability of findings across different telecommunications markets, regulatory environments, and customer populations. Future research should validate the proposed framework on multiple datasets from diverse operational contexts. The temporal constraint of single-timepoint data prevents analysis of customer behavior evolution and limits causal inference. The absence of external market factors, such as competitor pricing and regional availability, may affect generalizability across different telecommunications markets. Additionally, the focus on structured data omits potential predictive signals from unstructured sources like customer service interactions and social media sentiment.

### 5.3 Future research directions

Future research should address current limitations while building upon the established framework. **Multi-dataset validation:** Future studies should apply the proposed framework to additional telecom datasets from different geographical regions and market contexts to assess its generalizability and identify context-specific adaptations that may be necessary for optimal performance. Temporal modeling incorporating customer journey dynamics could enhance predictive accuracy and enable more nuanced intervention timing. Real-time prediction systems with streaming data integration would support proactive rather than reactive retention strategies. The integration of multi-channel data sources, including call center transcripts and social media interactions, could provide a more comprehensive customer view. Additionally, while this study conducted comprehensive clustering analysis using multiple algorithms (AE+KMeans, GMM, Spectral, DBSCAN) and identified three distinct customer segments with clear churn risk differentiation (15%–42%), the detailed methodology, extended validation metrics, and segment-specific intervention strategies will be presented in a separate publication due to space limitations.

The integration of causal inference methods could help transition from correlation-based insights to causal relationships, enabling more precise intervention design and resource allocation. The framework established in this research provides a robust foundation for these future advancements, demonstrating that combining advanced machine learning with business-centric explainability enables organizations to move beyond predictive accuracy toward actionable, data-driven customer retention strategies with measurable financial impact.

## 6 Conclusion

This research builds upon existing techniques by systematically comparing multiple machine learning approaches and integrating them within a business-aligned evaluation framework. Our findings confirm the effectiveness of gradient boosting algorithms for this dataset while demonstrating how model interpretability and cost-sensitive evaluation can enhance practical deployment. Specifically, the comprehensive framework for customer churn prediction successfully bridges the gap between machine learning performance and business applicability. The integrated approach combining multiple machine learning models, SMOTE oversampling and probability calibration yielded strong predictive performance. A key finding was that gradient boosting algorithms consistently outperformed other approaches, with XGBoost demonstrating the highest discriminative ability (AUC-ROC: 0.932) and balanced performance (F1-score: 0.84). The soft-voting ensemble of top models effectively consolidated their strengths, matching their F1-score performance (0.84) while maintaining robust discriminative ability (AUC-ROC: 0.918). Random Forest showed solid performance (AUC-ROC: 0.887, F1-score: 0.81) without evidence of the overfitting that might be indicated by perfect training metrics, providing a reliable alternative for certain deployment scenarios.

The systematic comparison revealed critical insights into model behavior: while gradient boosting algorithms (XGBoost, LightGBM, and Gradient Boosting) achieved the highest balanced performance, ensemble methods provided effective consolidation without sacrificing reliability. The integration of cost-sensitive evaluation with realistic telecommunications industry parameters further enhanced the framework's business relevance, with threshold effectively balancing precision and recall.

From a methodological perspective, this study extends existing work by providing a comprehensive evaluation of seven machine learning algorithms and one deep learning model, offering empirical evidence of performance variations in telecom churn prediction. The systematic comparison of clustering approaches revealed the superiority of autoencoder-based methods for high-dimensional customer data. Furthermore, the calibration and threshold optimization process demonstrated how technical model improvements directly translate to business value, enabling more accurate risk assessment.

The practical impact of this research is substantial, enabling a shift from reactive to proactive customer retention. The SHAP-based identification of contract type as the feature making the largest contribution to churn predictions, with month-to-month contracts associated with substantially higher predicted risk than 2-year contracts in the model, provides strategic direction that aligns with observed patterns. Similarly, the quantification of service bundle effects, showing significantly lower churn risk for customers with multiple premium services, offers empirical support for product development and bundling strategies. The customer segmentation analysis revealed three distinct risk profiles (15%, 21%, and 42% churn rates), enabling targeted resource allocation.

For industry practitioners, three key recommendations emerge: prioritize early intervention for high-risk segments identified through tenure and contract type analysis; expand service bundling strategies to increase customer stickiness; and integrate explainable AI techniques to build stakeholder trust and enable data-driven decision making. The framework's business alignment, through cost-sensitive evaluation and threshold optimization, strengthens the case for predictive analytics deployment in customer retention programs.

In summary, this research establishes that a multi-model framework, combined with business-centric explainability and cost-sensitive evaluation, enables organizations to transform predictive analytics into a strategic advantage. The findings not only highlight the superior performance of gradient boosting algorithms for churn prediction but also provide a practical pathway for deployment through robust models and interpretable insights. Future work should focus on dynamic customer journey modeling, multi-channel data integration, and causal inference methods to further enhance predictive accuracy and strategic value in real-world telecom environments.

## Data Availability

The original contributions presented in the study are included in the article/supplementary material, further inquiries can be directed to the corresponding author.
