# Supplementary material for: Explainable AI-driven customer churn prediction: a multi-model ensemble approach with SHAP-based feature analysis
Source: Front Artif Intell. 2026 Feb 10;9:1748799. doi: 10.3389/frai.2026.1748799 (PMC12929532; doi:10.3389/frai.2026.1748799)
Supplement: Supplementary file 1 [file Supplementary_file_1.pdf]

Appendix

January 30, 2026

A Autoencoder Architecture and Clustering Equations

A.1 Autoencoder Architecture

The autoencoder implements symmetric encoder-decoder transformations:

$$\text{Encoder: } \mathbf{z} = f_e(\mathbf{x}) = \sigma(\mathbf{W}_e\mathbf{x} + \mathbf{b}_e) \tag{1}$$

$$\text{Decoder: } \hat{\mathbf{x}} = f_d(\mathbf{z}) = \sigma(\mathbf{W}_d\mathbf{z} + \mathbf{b}_d) \tag{2}$$

where $\mathbf{x} \in \mathbb{R}^{46}$ is the input, $\mathbf{z} \in \mathbb{R}^{16}$ the latent representation, and $\hat{\mathbf{x}}$ the reconstruction.

Training minimizes the mean squared error with L2 regularization:

$$\mathcal{L}_{\text{AE}} = \frac{1}{N}\sum_{i=1}^{N} \|\mathbf{x}_i - \hat{\mathbf{x}}_i\|_2^2 + \lambda\|\mathbf{W}\|_2^2, \quad \lambda = 0.001 \tag{3}$$

Training parameters: Adam optimizer (learning rate 0.001), 100 epochs, early stopping (patience 15 epochs), batch size 32.

A.2 Clustering Equations

K-means clustering minimizes the within-cluster sum of squares:

$$\mathcal{J} = \sum_{i=1}^{N}\sum_{k=1}^{K} r_{ik}\|\mathbf{z}_i - \boldsymbol{\mu}_k\|^2 \tag{4}$$

where $r_{ik} = 1$ if $\mathbf{z}_i$ is assigned to cluster k (0 otherwise), $\boldsymbol{\mu}_k$ is cluster k's centroid, and K is the number of clusters.

Implementation details: feature standardization before clustering, K-means++ initialization, 10 random restarts with `random_state=42`, maximum 300 iterations.

Table 1: Autoencoder Architecture and Training Specifications

Component	Value	Description
**Architecture**		
Encoder Layers	46 → 32 → 16	Input (46), Hidden1 (32 ReLU + Dropout 0.2), Latent (16 ReLU)
Decoder Layers	16 → 32 → 46	Latent (16), Hidden1 (32 ReLU + Dropout 0.2), Output (46 Linear)
**Training Parameters**		
Optimizer	Adam	Learning rate = 0.001, $\beta_1 = 0.9$, $\beta_2 = 0.999$
Batch Size	32	
Epochs	100	
Early Stopping	Yes	Patience = 15 epochs, monitoring validation loss
Loss Function	MSE	Mean Squared Error with L2 regularization (λ = 0.001)
Random Seed	42	For weight initialization and training reproducibility
**Implementation**	Keras (v2.12.0)	TensorFlow backend (v2.12.0)

A.3 Cluster Validation Metrics

- **Silhouette Score**:
$$s(i) = \frac{b(i) - a(i)}{\max\{a(i), b(i)\}} \quad (5)$$
where $a(i)$ is the mean intra-cluster distance, $b(i)$ is the mean nearest-cluster distance.

- **Calinski-Harabasz Index**:
$$\text{CH}(K) = \frac{\text{trace}(\mathbf{B}_K)/(K-1)}{\text{trace}(\mathbf{W}_K)/(N-K)} \quad (6)$$
where $\mathbf{B}_K$ is between-cluster dispersion, $\mathbf{W}_K$ is within-cluster dispersion.

- **Davies-Bouldin Index**:
$$\text{DB}(K) = \frac{1}{K}\sum_{i=1}^{K} \max_{j \neq i} \left(\frac{\sigma_i + \sigma_j}{d(c_i, c_j)} \right) \quad (7)$$
where σ_i is the average distance to centroid c_i, $d(c_i, c_j)$ is the distance between centroids.

B Hyperparameter Search Details

B.1 Search Methodology

All hyperparameter optimization used grid search with 5-fold stratified cross-validation, optimizing F1-score. The search was conducted on the training set only, with the test set reserved for final evaluation.

B.2 Search Spaces by Model

Table 2: Hyperparameter search spaces for all models

Model	Search Space and Strategy
XGBoost	Grid search: `learning_rate` [0.01, 0.1, 0.3], `max_depth` [3,6,9], `n_estimators` [50,100,200], `subsample` [0.6,0.8,1.0], `colsample_bytree` [0.6,0.8,1.0]
Random Forest	Grid search: `n_estimators` [50,100,200], `max_depth` [None, 10, 20], `min_samples_split` [2,5,10]
LightGBM	Bayesian optimization (50 iterations): `num_leaves` (20,40), `learning_rate` (0.01,0.3), `feature_fraction` (0.6,1.0)
MLP	Random search (30 iterations): `hidden_layer_sizes` [(64,32), (128,64), (64,32,16)], `alpha` [0.0001, 0.001], `batch_size` [16,32,64]

B.3 Optimization Results

Final hyperparameters were selected based on cross-validated F1-score. All optimization procedures maintained a fixed random state (42) for reproducibility.
